# Early and Unintentional Release of Planned Motor Actions during Motor Cortical Preparation

**DOI:** 10.1371/journal.pone.0063417

**Published:** 2013-05-07

**Authors:** Colum D. MacKinnon, David P. Allen, Takako Shiratori, Mark W. Rogers

**Affiliations:** 1 Department of Neurology, University of Minnesota, Minneapolis, Minnesota, United States of America; 2 Department of Physical Therapy and Human Movement Sciences, Feinberg School of Medicine, Northwestern University, Chicago, Illinois, United States of America; 3 Department of Physical Therapy and Rehabilitation Science, Departments of Neurology and Anatomy and Neurobiology, University of Maryland School of Medicine, Baltimore, Maryland, United States of America; Florey Institute of Neuroscience & Mental Health, Australia

## Abstract

Voluntary movements are often preceded by a movement-related potential beginning as much as two seconds prior to the onset of movement. In light of evidence that motor actions can be prepared and initiated in less than 200 ms, the function of this early activity has remained enigmatic. We hypothesized that the movement-related potential reflects the state of preparation of the planned movement. This was tested by delivering a startling acoustic stimulus during the preparation phase of a load-release task. The cue to release the load was presented either 3.5 seconds after a warning cue (PREDICT condition) or randomly between 4–12 seconds (REACT condition). Electroencephalographic, electromyographic and limb and load kinematic signals were recorded. In a subset of trials, a startle stimulus was delivered at −1500, −1000, −500, −250, −100 or 0 ms before the release cue. A contingent-negative variation (CNV) waveform, with a late phase of slow-rising negativity beginning an average of 1459 ms prior to movement, was observed for the PREDICT condition but not the REACT condition. For both conditions, the startle stimulus frequently evoked the early and unintentional release of the load-release sequence. The incidence of release was significantly (p<0.001) correlated with the late phase of the CNV for the PREDICT condition but not the REACT condition. For the REACT condition, the incidence of movement release was subject-specific, constant across the preparation interval, and uncorrelated with cortical activity. The onset of movement release by the startle stimulus was significantly shorter (p<0.001) for the PREDICT compared to the REACT condition. These findings provide evidence that the late phase of the CNV reflects cortical activity mediating the progressive preparation and storage of the forthcoming movement and that during this phase an intense sensory stimulus can evoke early and unintentional release of the planned action.

## Introduction

Our environment is filled with sensory cues that influence the timing and expression of voluntary actions. Behaviorally relevant cues often require a reaction to the stimulus (reactive movements) or provide advance information that can be used to proactively plan the timing and content of the forthcoming actions (predictive movements). In the latter context, movements are often preceded by a slow-rising increase in cortical activity beginning as much as 2–3 seconds before movement onset [1]–[3]. This early cortical activity can be measured using scalp surface electroencephalographic (EEG) recordings back-averaged relative to the onset of muscle activity. When the movement is initiated under self-paced conditions, the movement-related EEG waveform is termed the “Readinesss” or Bereitschaftspotential (BP) [2] and when it is initiated in response to an imperative stimulus that is preceded by a warning stimulus, the waveform is termed the Contingent Negative Variation (CNV) [3]. The CNV is characterized by two components: an early period of prolonged negativity that immediately follows the potential evoked by the warning stimulus, followed by a late component that is distinguished by a slow-rising negativity that is time-locked to movement onset with maximal amplitude over motor regions of the frontal cortex. The late component of the CNV is also accompanied by a progressive suppression of oscillations in the alpha (8–12 Hz) and beta-bands (13–30 Hz), termed event-related desynchronization [4], that is considered to reflect a transition from an idling motor state to a state of readiness to act [5], [6]. In light of the fact that voluntary movements can normally be initiated in less than 200 ms, the function of this early ramping motor cortical activity has remained enigmatic [7]. It has been proposed that early preparatory activity reflects the progressive selection and construction of the spatial and temporal components of the planned movement and thus represents a subthreshold form of the motor output which allows for faster reaction times and reduced movement variability [8]–[11]. More recently, it has been proposed that preparatory activity reflects the initial state of a dynamical system in which the early neuronal population activity does not represent the forthcoming movement parameters [12]. Resolution of this problem is greatly limited by the inability to decode the function of movement-related preparatory activity, whether obtained from single-, multi-unit or local field potential recordings of neuronal activity in behaving non-human primates or scalp surface recordings from humans, and its relationship to the eventual motor output.

In this paper, we implemented a novel paradigm that uses a startling acoustic stimulus (SAS) to probe the state of preparation of the motor output. This paradigm was derived from the seminal experiments first described by Valls-Solé et al. [13], [14] showing that voluntary movement sequences could be rapidly released, at latencies of less than 100 ms, when an intense acoustic stimulus (130 dB) was presented in conjunction with the imperative cue to initiate movement; a phenomenon termed the “StartReact”. Two salient features of the StartReact are: (1) despite the extraordinarily short reaction times, the spatial and temporal features of the planned movement sequence remain unchanged and (2) the rapid initiation of the intended movement only occurs when the movement to be performed is known in advance [15]. Based on the short latency of these reactions, it has been proposed that the StartReact reflects a reflexive or “involuntary” release of the planned movement via fast conducting pathways [13], [15]–[17]. Consistent with this idea, it has been shown that movements can sometimes be released by a startling stimulus presented well before the imperative go-cue, however the incidence of release was related to the timing of presentation of the startle stimulus and temporal resolution of the delay interval between a warning and go-cue [18], [19]. Since cortical preparation is known to be modulated by the temporal predictability and resolution of the imperative cue [20]–[23], these findings suggest that the capacity of an intense sensory stimulus to evoke a StartReact might be dependent upon the timing and level of motor cortical preparation. Our study tested this hypothesis by recording scalp surface electroencephalographic signals (EEG) during the preparation and execution of a task performed under temporally reactive or predictive conditions.

## Materials and Methods

### Subjects

Nine right-handed neurologically healthy subjects (9 males; average age  =  33, range  =  27 to 49 years) participated in these experiments. Hand dominance was assessed using the Edinburgh Handedness Scale [24]. The experiments were approved by the Institutional Review Board at Northwestern University and written informed consent was obtained prior to inclusion into the study.

### Experimental design

#### Task

**Figure 1:**
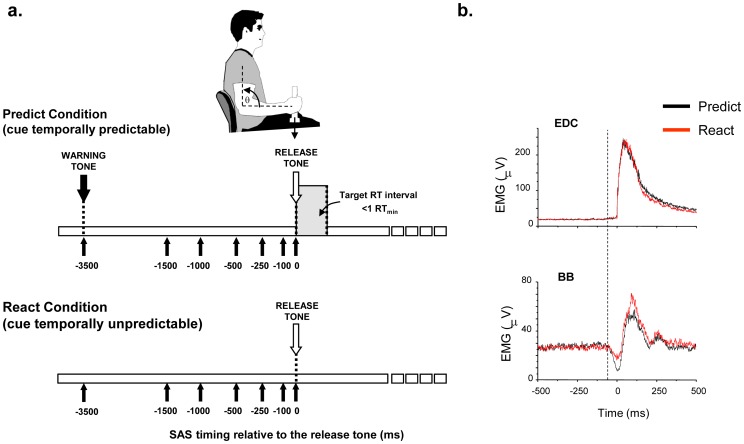
Summary of the stimulus timing conditions and muscle activation patterns. A load release task was tested using two cueing conditions: PREDICT and REACT. A. Summary of the timing of the cues and presentation of the startle stimuli. For the PREDICT condition, subjects were presented with a warning tone 3500 ms prior to an imperative cue release tone. Subjects were trained to release the load within 1 simple reaction time in 50% of trials. For the REACT condition, there was no warning cue and the release tone was presented randomly between 4–12 s. For both conditions, a 40 ms, 124 dB startling acoustic stimulus (SAS) was presented in 22% of all trials at 7 time points over the preparation interval. B. Average rectified EMG data from a single subject showing responses in the biceps brachii (BB) and extensor digitorum communis (EDC) muscles during the PREDICT and REACT conditions. EMG responses were time-locked to the onset of activity in the EDC muscle (0 ms).

Subjects sat in a chair and held a vertical rod attached to a 1.25 lb (0.57 kg) load with their shoulder abducted to approximately 20 deg., forearm parallel to the ground and elbow flexed at 90 deg (Figure 1). The task required subjects to release the load “as fast as possible” in response to an acoustic (80 dB) imperative cue. They were further instructed to maintain a stationary posture of the elbow and shoulder prior to, during and after release of the load. Correct performance of this task requires a stereotypical sequence of anticipatory postural adjustments (initial inactivation of elbow flexor muscles, followed by finger and wrist extensor activity) to ensure that elbow posture remains stable during the instructed focal movement of the wrist and fingers [25]. This task was chosen to ensure a clear differentiation between muscle activities associated with the intended movement sequence from that of a classic startle reflex (typically associated with rapid elbow flexion).

Subjects performed the load release task under two different cue timing conditions: (1) an instructed-delay condition with fixed timing between a warning and imperative “release” cue (PREDICT condition) and (2) a randomly timed imperative “release” cue (REACT condition). The PREDICT condition used an initial warning tone (50 ms, 80 dB) followed 3.5 s later by a “release” tone (50 ms, 80 dB). Subjects were trained to initiate the load release movement within less than one simple reaction time in 50% of trials (see ref. [20] for details of this protocol) but were explicitly instructed to avoid initiating movement prior to the imperative cue. This constraint ensured that subjects predicted, rather than reacted, to the onset of the tone but did not initiate within the preparation interval. This paradigm has been shown to be associated with an early and large amplitude CNV waveform [20], [26]. For the REACT condition there was no warning tone and the imperative “release” tone was presented randomly between 4 and 12 s. Under this condition, subjects can only react to the imperative cue and a CNV is normally absent [20], [26]. Thus, these two tasks allowed comparison of movement response properties with and without an early build-up of movement-related EEG activity. Eight blocks of 24 trials were performed for each task (192 trials).

### Startling Acoustic Stimulus (SAS) to probe the state of preparation

In a subset of trials a startling acoustic stimulus (SAS) (40 ms, 1000 Hz, 123 dB) was pseudo-randomly presented at one of six different time points prior to the imperative cue (−1500, −1000, −500, −250, −100, 0 ms; 6 trials at each time point). In addition, 6 trials were collected in which SAS was presented at −3500 ms and no warning or imperative tone was provided (SAS-only trials). SAS was not presented in the first 2 trials of any block and there were never two consecutive startle trials.

### Data acquisition

**Figure 2:**
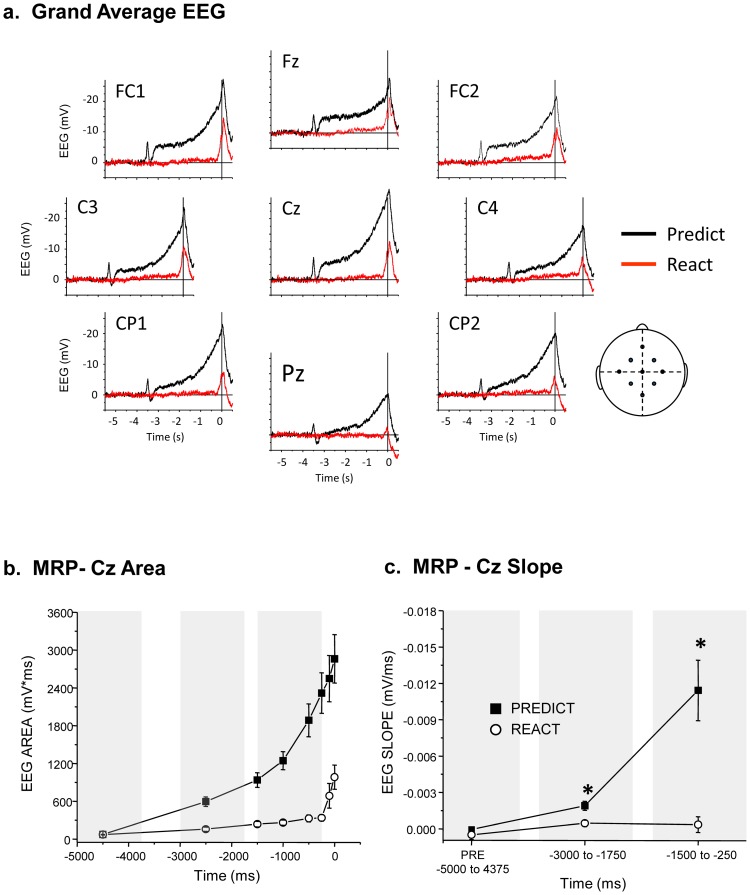
Movement-related potentials (MRPs) were affected by the predictability of the imperative release cue. A. Grand average movement-related potentials derived from control trials at each of the nine EEG electrodes for both the PREDICT (black) and REACT (red) conditions. The cartoon to the right shows the locations of the electrodes on the scalp. B. Average integrated EEG calculated over 100 ms time bins at 8 time points centered on −4500, −2500, −1500, −1000, −500, −250, −100, 0 ms. Differences between conditions were significant (p<0.007) at all time points except at baseline (−4500 ms). C. Average slope of the movement-related potentials over three time intervals. The slopes were significantly different between conditions during movement preparation but not baseline (PRE) (p<0.007).

Scalp surface EEG was recorded (band-pass filter: DC - 250 Hz) from a montage of 9 electrodes (see Figure 2) placed according to standard 10–20 locations (C3, Cz, C4, Pz, P3, P4, FC3, Fz, FC4) and referenced to linked mastoid electrodes. Electroculography (EOG) was recorded using electrodes placed over the upper and lower canthi of one eye. EEG and EOG signals were collected at 1000 Hz (Synamps, Scan 4, Compumedics, Neuroscan). EMG recordings were obtained from surface electrodes over the motor points of the right extensor digitorum communis, biceps brachii and sternocleidomastoid (a startle indicator) muscles. Acceleration signals were obtained from a uniaxial accelerometer (Entran, Model EGCS-D1S) attached to the dorsum of the intermediate phalange of the third finger. Elbow joint position was collected using an electrogoniometer (Biometrics, Ltd.) aligned to the flexion/extension axis of the elbow. Electromyographic (EMG) activity was recorded (gain  =  2000, band-pass filter  =  30–1000 Hz) from bipolar surface electrodes placed over the motor point of the extensor digitorum communis (EDC), biceps brachii (BB) and sternocleidomastoid (SCM). All EMG, acceleration and joint displacement data were collected at 2000 Hz (Power 1401, Signal 3, CED Ltd.

### Data processing

Reaction times to the imperative cue or to the SAS were calculated based on the onset of the rectified EMG signal in EDC. EMG onset was defined as the time point when the rectified EMG exceeded baseline levels by more than three standard deviations for more than 40 ms. Baseline EMG levels were measured from 100 to 0 ms prior to the release cue for control trials and from 100 to 0 ms prior to the SAS for SAS trials. The reaction time was then inspected and adjusted manually to coincide with the initial rise of the EMG burst [27]. A similar algorithm was used to detect the onset and offset of anticipatory EMG suppression in the biceps muscle. The kinematic variables quantified were the peak angular displacement and velocity of the elbow following load release and onset of the hand extension acceleration.

EEG data was epoched into single movement segments from −5500 to +500 ms relative to the onset timing of EDC EMG activity. Epochs were DC corrected and trials containing eye movements, blinks, or other artifacts were rejected before averaging across trials. The magnitude of the premovement potentials were quantified by calculating the average integrated signal across six times bins (each bin  =  ± 50 ms) centered on −4500, −2500, −1500, −1000, −500, −250, 100 and 0 ms. Frequency domain processing was performed with MATLAB® Version 7.5 (The MathWorks, Inc., Natick, MA). Epoched data were low-pass filtered (dual-pass 2^nd^-order Butterworth filter, 62.5 Hz cut-off) and down-sampled by a factor of 4 to give an effective sampling rate of 250 samples/s. Epochs were subsequently high-pass filtered to remove DC offset and drift (dual-pass 2^nd^-order Butterworth, 1 Hz cut-off). Spectrograms for each filtered epoch of electrode C3 were generated using the Short-time Fourier Transform (STFT) with a window length of 0.5 s (i.e., 125 samples) and 124 sample overlap. A mean spectrogram for each subject and condition (PRECICT and REACT) was obtained by averaging across epochs. Normalized spectrograms were referenced to a period prior to the release cue ranging from −5.0 to − 4.25 s. Statistical comparisons of the spectrograms between baseline, early (−3000 to −2000 ms) and late (−1500 to −500 ms) preparation were conducted using the method of Diggle [28]. The time courses of alpha and beta power were obtained by averaging spectrogram power across subjects in the bands spanning 8 to 12 Hz and 18 to 22 Hz, respectively. Mean values across subjects for each condition were calculated across time bins (±50 ms) centered on −4500, −2500, −1500, −500, −250, −100, and 0 ms.

### Statistical analysis

Dependent variables were analyzed using repeated measures ANOVAs for the factors of Task Condition (PREDICT vs. REACT) and SAS timing (−3500, −1500, −1000, −500, −250, −100, 0 ms). Incidence variables were converted using an arcsine square root transform prior to analysis. Greenhouse-Geisser corrected degrees of freedom were used to correct for violations of the assumption of sphericity. Tukey's Honestly Significant Differences (HSD) tests were conducted for post-hoc examination of interaction effects. Differences between conditions in SAS-evoked reaction times were analyzed using a Wilcoxon Signed Ranks Test. The relationship between movement-related potentials and incidence of release of movements by SAS was examined using a linear regression analysis. Differences or effects between variables were considered to be significant at the p<0.05 level.

## Results

### Task performance

Task performance for both cue conditions was characterized by an initial suppression of biceps brachii activity (duration of silence: PREDICT  =  96 ± 42 ms, REACT  =  83 ± 50 ms) followed by activation of the wrist extensor muscles (Figure 1B). There were no significant differences in the relative timing (onset of BB silence to onset of EDC burst), duration or magnitude of the BB silence and EDC burst for control (non-SAS) trials across tasks (p>0.16). As expected, reaction times for the PREDICT condition (116 ± 8 ms) were significantly faster than the REACT condition (185 ± 17 ms) (p = 0.012). The shorter reaction time for the PREDICT condition is consistent with subjects attempting to predict the temporal onset of the release cue.

### Temporally predicted movements are preceded by preparatory cortical activity

**Figure 3:**
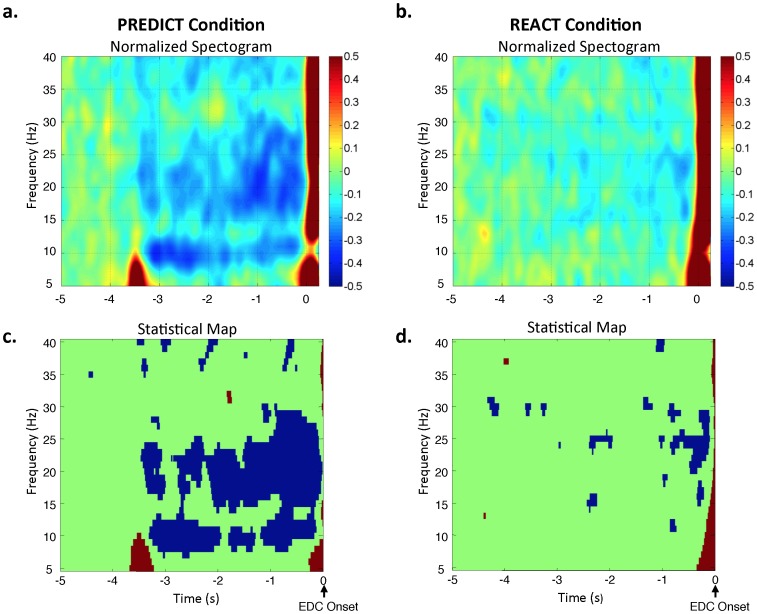
Movement-related oscillations were affected by the predictability of the imperative release cue. The upper plots show the grand average time-frequency spectrograms at the C3 electrode overlying the contralateral sensorimotor region for the PREDICT (A) and REACT (B) conditions. Magnitudes have been normalized to the power over the pre-warning cue interval (−5 to −4.25 s). Note the marked suppression of oscillations in the alpha band (8–12 Hz) immediately following the warning cue and in the beta-band (15–30 Hz) during the 1500 ms time period immediately preceding the onset of wrist extensor (EDC) muscle activity (0 ms) for the PREDICT task. In contrast, the suppression of MRCOs occurred immediately prior to EMG onset for the REACT task and was confined to the beta-band. Plots C and D show the results of the statistical analysis of the normalized time-frequency spectrograms at the C3 electrode for the PREDICT (C) and REACT (D) conditions. Significant (p<0.05) increases (red) and decreases (blue) in MRCOs relative to baseline activity are shown.

In contrast to task performance, the time course of cortical preparation was markedly different between conditions (Figure 2). The PREDICT condition was associated with a CNV waveform characterized by an initial auditory-evoked potential in response to the warning tone at −3500 ms, a subsequent period of sustained activity with a slow developing negative slope, followed by a more rapid-rising negative potential beginning an average of 1459 ± 320 ms (mean ± 1 stdev) prior to the onset of wrist extensor muscle activity (Figures 2B and 2C). The movement-related potential for the REACT condition was associated with a very shallow slope throughout the preparation interval, followed by an abrupt increase in negativity beginning an average of 150 ± 68 ms prior to EMG onset (117 ms after the release cue). Significant interaction effects of Task Condition × SAS Timing (F 17.1, p<0.001) were observed for both the magnitude and slope of the movement-related potential at Cz. These interactions reflected a significant increase in the magnitude and slope for the PREDICT relative to the REACT condition at all time points with the exception of the baseline time period (−5000 to −4000 ms) (Figure 2B and C). The PREDICT condition was also associated with a suppression of movement-related oscillations in the alpha and beta bands over the contralateral sensorimotor cortex (Figure 3) during the delay interval between the warning and release cues. Oscillations in the alpha band were significantly suppressed (95% confidence intervals) during the time interval immediately following the warning cue (−3 to −2 s prior to EMG onset) in 6 of 9 subjects whereas late in the preparation interval (−1.5 to −0.5 s prior to EMG onset) only 2 subjects showed a significant suppression. In contrast, beta oscillations were significantly suppressed late in the preparation interval (6 of 9 subjects), but less commonly immediately after the warning cue (3 subjects). Oscillations for the REACT task were characterized by little change in the magnitude of alpha oscillations throughout the preparation interval (a significant suppression was observed in only 1 subject) while beta oscillations were suppressed approximately 1500 ms prior to movement onset, but this suppression was significant in only 1 subject.

### SAS evokes the unintentional release of the movement

**Figure 4:**
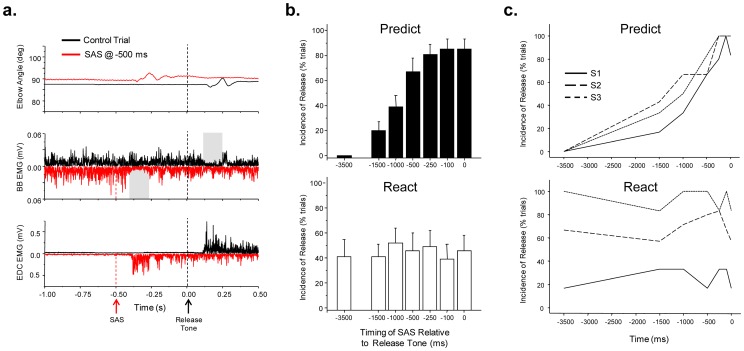
Summary of the effects of a startling acoustic stimulus (SAS) on the release of movement. A. Single trial examples of the elbow joint displacement and rectified EMG activity in biceps brachii (BB) and extensor digitorum communis (EDC) muscles when movement was initiated in response to the imperative tone (control trial, black line) or in response to a SAS presented 500 ms before the release tone (red line). Note that the kinematics and muscle activation patterns (the BB EMG suppression is highlighted with a grey bar) for the SAS-evoked movement were unchanged from control trials but the sequence was initiated less than 100 ms after the startle stimulus. B. The incidence of release of the movement sequence by a SAS was significantly affected by the temporal probability of imperative cue. For the PREDICT condition, the average incidence of release progressively increased from 0% of trials at −3500 ms (warning cue) to over 80% at −100 and 0 ms. In contrast, the incidence of release was relatively constant across all stimulus timings for the REACT condition. C. Examples of the incidence of movement release as a function of the timing of the SAS across three subjects (S1, S2, S3). For the PREDICT task, all subjects showed a similar profile of increasing incidence of release. In contrast, the behavior for the REACT task was different across subjects. S1 rarely released, S3 released on nearly every trial and S2 released in approximately 60% of trials, irrespective of timing of the SAS.

The presentation of a SAS during the movement preparation phase for both task conditions frequently resulted in the spontaneous and rapid extension of the fingers and release of the load (Figure 4A). Nonetheless, subjects maintained the required posture of the limb by preceding finger extensor activity by a suppression of muscle activity in the biceps brachii muscle. Subjects often commented that they were unable to withhold the action, despite explicit instructions to avoid initiating the task before the release cue. However, the two task conditions differed markedly with respect to the incidence and latency of spontaneous release of the load (Figure 4B). For the PREDICT condition, there was a significant main effect of SAS timing (F_1,6_ = 37.34, p<0.001) on the incidence of load release, reflecting a progressive increase in the probability of spontaneous release of the movement. The incidence of release of the load increased from a mean of 20% of trials for SAS at −1500 ms, to over 80% of trials for SAS at −100 or 0 ms. When SAS was presented at the time of the warning cue (−3500 ms), subjects never spontaneously dropped the load. There was a significant linear relationship (r^2^ = 0.937; p<0.001) between the incidence of release of movement by SAS and the magnitude of the CNV (Figure 4C, top plot). In contrast, there was no significant main effect of SAS timing for the REACT condition (F_1,6_ = 0.62, p = 0.71) since the mean incidence of release of the load remained relatively constant across all time intervals, including the SAS-only (−3500 ms) trials. However, there was considerable variation in the behavior across subjects for the REACT condition; some subjects consistently dropped the load in response to the SAS, while others rarely dropped the load (Figure 4C, bottom plot). Nonetheless, the incidence of load release remained relatively constant across all timing intervals within a subject. There was no significant relationship between the magnitude of the movement-related potential and incidence of release of the movement by SAS for the REACT trials (r^2^ = −0.012, p = 0.82).

### SAS-evoked movements are rapidly released

**Figure 5:**
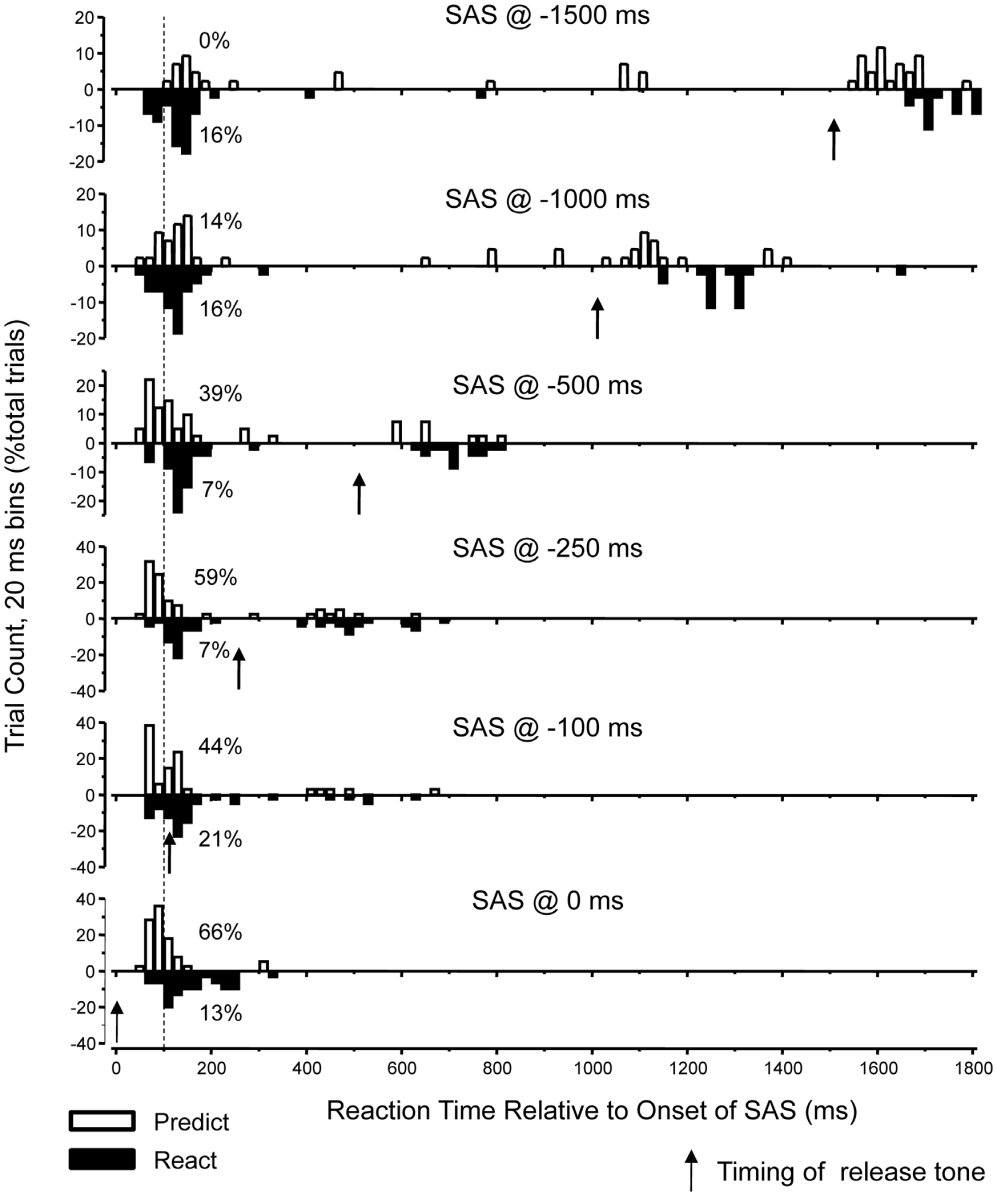
Summary of the effects of a startling acoustic stimulus (SAS) on the timing of movement release. Reaction times relative to the onset of the SAS were sorted into 20 ms bins. The histograms show the percentage of trials within each reaction time bin for both the PREDICT (open rectangles) and REACT (filled rectangles) tasks. The vertical black arrows show the timing of the imperative release tone. The vertical dashed line is drawn at reaction time of 100 ms. The percentage of trials with reaction times less than 100 ms is shown to the right of the histogram distributions. Note that fast reaction times were common for the PREDICT condition when the SAS was presented 250 ms or less before the imperative cue.

Both the task condition and timing of SAS had a significant effect on the speed of release of the load in response to the startle stimulus. Figure 5 shows the distribution of reaction times when SAS was presented during the preparation interval. For the PREDICT condition, presentation of a SAS at −500, −250, −100 or 0 ms resulted in the rapid release of the movement task with median reaction times of 114, 97, 103 and 91 ms respectively. When a SAS was delivered at earlier time points (−1500, −1000 ms) fewer trials were associated with rapid release of the movement and a smaller percentage of trials had reaction times to SAS of less than 100 ms. In contrast, for the REACT condition, the median reaction times remained relatively constant across SAS timing conditions (−SAS_−1500_  =  158 ms; SAS_−1000_  =  146 ms; SAS_−500_  =  156 ms; SAS_−250_  =  161 ms; SAS_−100_  =  137 ms; SAS_0_  =  141 ms) and onsets of less than 100 ms were much less common compared to the PREDICT condition. SAS-induced reaction times were significantly faster for the PREDICT compared to the REACT condition when a SAS was provided at −500, −250, −100 and 0 ms (p<0.001).

## Discussion

Our findings demonstrate that, under preparatory conditions in which the timing of onset of the imperative cue could be predicted in advance, the presentation of a SAS could rapidly and unintentionally release a planned movement sequence well in the advance of the intended time of action. However, the incidence and latency of release of the movement by a SAS was critically dependent on the timing of the startling stimulus, the temporal predictability of the impending imperative go-cue and the associated movement-related potential.

It has been proposed that preparatory neuronal activity, and the associated scalp surface EEG signal, reflects a progressive facilitation of cortical and subcortical pathways contributing to the kinematic, kinetic and temporal components of the forthcoming movement initiation sequence [8]–[11], [29], [30] such that the system moves closer to a triggering threshold in expectation of the incoming sensory volleys evoked by the imperative cue (the subthreshold hypothesis). More recently, this idea has been challenged based on data showing that preparatory activity at the neuronal population level does not represent specific biomechanical features of the forthcoming movement, but instead represents the initial state of a dynamical system that is optimized to drive the initiation of movement during the immediate peri-movement phase [12]. In many respects, the results of the present study support the subthreshold hypothesis. The fact that movement sequences could be evoked by a SAS as early as 1.5 s prior to the expected cue, and at latencies of less than 100 ms, demonstrates that the spatial and temporal features of the initial motor response were fully prepared and could be released when an initiating trigger brought the system past threshold. The low incidence of movement release early in preparation could be explained by a high threshold for triggering, but also by an active suppression of motor output at the cortical and spinal motor neuron levels [31], [32]. This early suppression of excitability has been proposed as a mechanism of “impulse control” that prevents the premature release of the intended response [31]–[33]. The progressive increase in the incidence of movement release and decrease in the response latency in response to SAS over the preparatory interval is consistent with the idea that impulse control is suppressed, the movement initiation threshold is decreased, the rate of rise to threshold is increased [9] and variability in the state of preparatory neural activity is decreased [34]. Thus, the slow-rising CNV beginning near 1500 ms prior to movement onset, and accompanying suppression of movement-related oscillations, could best described as a progressive “releasing of the brakes” [35] or “opening of the gates”, rather than activity associated with higher-order aspects of motor preparation such as attention and planning.

Late in preparation (less than 500 ms), there was little change in the incidence or timing of release of movement by the SAS. During self-initiated (uncued) movements, this phase of preparation is associated with an increase in the negative slope of the movement-related potential and a shift in topography towards the contralateral motor cortex (termed the BP2 or NS' phase) [1], [7], [29]. This change is often less prominent in the CNV waveform and could not be objectively distinguished from the early increase in negativity beginning near −1500 ms in this study. Nonetheless, our findings suggest that the state of preparation during this interval, in terms of readiness for release, has plateaued. This interpretation is consistent with recent work showing that incidence and timing of release of a simple wrist extension movement were unchanged when the SAS was presented 500 ms or less prior to the go-cue during a fixed foreperiod (3 s) and variable foreperiod (2–3) reaction time task [18].

In contrast to the PREDICT condition, the presentation of a SAS prior to the release cue for the REACT condition was associated with a near-constant within-subject probability of movement release by the intense stimulus. Under this condition, movement-related potentials were markedly reduced, the suppression of oscillations was attenuated, and SAS-evoked reaction times of less than 100 ms were significantly less common compared to the PREDICT condition. The grand average movement-related potentials and oscillations did exhibit a shallow slope, suggesting that some subjects were progressively preparing for the imperative cue, despite the temporal uncertainty of the required response. This low level preparatory activity was likely due to the fact that the maximum interval between trials was fixed at 12 s, thus the probability of presentation of the imperative cue increased as the duration of the inter-trial interval increased [36], [37]. However, the relatively stable incidence of SAS-evoked movement release within-subjects suggests that, under conditions of uncertainty about the timing of the imperative cue, the state of motor readiness and the sensorimotor threshold required to release the planned movement was held relatively constant throughout the inter-trial interval.

Due to the unusually short response times associated with the StartReact, Valls-Solé and others [13], [15], [17] hypothesized that movements evoked by a SAS were released involuntarily. Yet in those studies, the SAS was presented concurrently with the imperative go-cue, a time point in which the initiation of movement is considered to be intentional [38]. Voluntary behavior has been broadly defined as those actions that are conscious and suppressible [39]. In the present study, we found that movements could be evoked by a SAS as early as 1500 ms prior to the intended onset and the release of movement was often described as “involuntary” or “unintentional”. Late in preparation (less than 500 ms) movement release was predominantly insuppressible. The conscious experience of the intention to act is considered to arise from early processes related to the preparation for movement [38], [40], [41]. Libet and colleagues were the first to present evidence that motor preparation begins well in advance of the feeling of the urge to act [41]. Although our experiment was not designed to estimate the time point at which the release of movement by a SAS was considered to be intentional, our findings are consistent with the idea that fully formed movement sequences can be rapidly and involuntarily released by a SAS when the stimulus is presented during movement preparation.

These findings have important ramifications to motor performance. For example, the timing predictability of the starter's pistol discharge and the athlete's capacity to predict its onset will have a significant effect on their reaction times. If the athlete accurately predicts the onset of discharge they are much more likely to initiate movement with a reaction time of less than 100 ms, but if they prepare too late (i.e. the stimulus is presented early in preparation) or merely react to the sound, the reaction time is likely to be much slower. In sports where the difference between medalling and not medalling can be measured in hundredths of a second, the capacity to appropriately prepare can make all the difference.
